# Systematic Survey and Analysis Reveal Jasmonate ZIM-Domain Gene Family in *Coix lacryma-jobi* Under High Temperature

**DOI:** 10.3390/plants13223230

**Published:** 2024-11-17

**Authors:** Zhenming Yu, Yufeng Shen, Yiming Sun, Zhangting Xu, Feixiong Zheng, Xiaoxia Shen

**Affiliations:** 1 School of Pharmaceutical Sciences, Academy of Chinese Medical Sciences, Zhejiang Chinese Medical University, Hangzhou 310053, China; 20221012@zcmu.edu.cn (Y.S.); 20221013@zcmu.edu.cn (Y.S.); 15168104031@163.com (Z.X.); feixiongz@163.com (F.Z.); 2 Songyang Institute, Zhejiang Chinese Medical University, Lishui 323400, China

**Keywords:** *Coix lacryma-jobi*, JAZ, transcriptome, high-temperature, ABA

## Abstract

Jasmonate ZIM-domain (JAZ) acts as the repressor of the JA signaling pathway and plays a significant role in stress-inducible defense, hormone crosstalk, and the regulation of the growth-defense tradeoff. The aim of this study is to systematically survey and analyze the JAZ gene family in *Coix lacryma-jobi* and unveil its expression profiles in diverse organs under high-temperature stress using transcriptome. The results identified a total of 20 JAZ family proteins randomly mapped on four chromosomes and encoding 159–409 amino acids. They were clustered into six groups and were mainly located in the nucleus. The conserved motifs, gene composition, and secondary structure of ClJAZ members within the same subtribes were similar. Multitudinous *cis*-regulating elements employed in hormone responsiveness and stress responsiveness were displayed before the promoter sequences of *ClJAZ1*-*ClJAZ20*. *ClJAZ1*-*ClJAZ20* were differentially distributed across diverse organs (the roots, shoots, leaves, kernels, glumes, and flowers), exposed to high-temperature stresses, and treated using ABA or MeJA. A total of 29115 DEGs were identified under heat stress, which were mainly involved in biological regulation and the metabolic process. Intriguingly, *ClJAZ15* was highly expressed in the leaves of *C. lacryma-jobi*, down-regulated by MeJA, but up-regulated by heat stress and ABA, inferring that *ClJAZ15* might be associated with ABA-inducible heat stress. The results laid a foundation for in-depth study of the role of ClJAZ family genes in *C. lacryma-jobi*.

## 1. Introduction

Plants have evolved increasingly complex and ever-changing adaptations in response to surrounding abiotic stresses, for instance, heat, drought, and salinity [1]. Methyl jasmonate (MeJA) is a volatile hormone that can modulate various physico-biochemical processes such as stomatal movement, root development, and specialized metabolite accumulation, thereby mediating plant stress resistance [2]. It plays an important role in abiotic stress (drought, low temperature, heat, salinity, flooding, and exposure to heavy metals) and biotic stress (pathogen infection, insect attacks, and invasive weeds) [3,4]. Jasmonate ZIM (zinc-finger inflorescence meristem) domain (JAZ) proteins are critical regulators of the JA-signaling pathway, which uniquely exists in plants and belongs to the TIFY transcription factor (TF) with conserved functional domain (TIF[F/Y]XG). Based on the evolutionary characteristics of the TIFY superfamily and protein domain, it is classified into four subfamilies: TIFY, JAZ, peapod (PPD), and ZIM-like (ZML) [5]. The JAZ subfamilies are the most deeply studied among the TIFY superfamily, harboring both the TIFY and JAS domains [6]. JAZ members work as repressors targeting downstream JA-responsive genes, which dynamically influence growth and accurately cope with environmental stresses [7]. Hence, JAZ proteins can interact with multiple TFs or target genes through their transcriptional regions and play an indispensable role in regulating plant growth and development, hormone stimulation, and stress responsiveness.

Currently, the JAZ gene family has been widely characterized in monocots and dicots, including *Arabidopsis thaliana* [8], *Oryza sativa* [9], *Solanum lycopersicum* [10], *Nicotiana tabacum* [11], *Capsicum annuum* [12], and *Saccharum spontaneum* [13]. Different JAZ family members have diverse biological functions, especially in the JA signaling pathway [14]. In the absence of JA, JAZ interacts with Myelocytomatosis 2 (MYC 2), thereby generating the JAZ-MYC2 modulate, which inhibits the expression of JA-responsive genes [7,15]. While in the presence of JA, JAZ interacts with coronatine-insensitive 1 (COI 1), a JA signaling receptor, thereby generating the JAZ-COI 1 complex, which degrades JAZ, releases MYC2 from the JAZ-MYC2 complex, and activates the expression of JA-responsive genes [16]. Additionally, JAZ usually takes part in the differential accumulation of specialized metabolites, which is conducive to improved stress response and tolerance [17]. Despite the momentous role of the JAZ protein in plant vegetative and reproductive growth, there is still no information on the identification and expression profiles of JAZ family members in the medicinal and food-homogeneous plant *Coix lacryma-jobi*.

*C. lacryma-jobi*, also called adlay or Job’s tears, is an annual cereal crop whose kernels are used pharmaceutically and eaten [18]. It is abundant in polysaccharides, lipids, flavonoids, phenols, vitamins, and other bioactive ingredients, which are essential to anti-tumor, antibacterial, antiviral, anti-inflammatory, analgesic and antioxidant activity and the regulation of blood lipid effects [19,20]. During the growth and development of *C. lacryma-jobi*, it is usually faced with various stresses, and its specialized metabolites are influenced through the JA signaling pathway. Herein, a genome-wide characterization of the *C. lacryma-jobi* JAZ gene family was implemented based on the chromosome level of the *C. lacryma-jobi* genome. The physicochemical properties, conserved motifs, evolutionary relationships, *cis*-regulating elements, and chromosomal localization of the gene family were systematically investigated. The expression profiles of JAZ family genes in different *C. lacryma-jobi* tissues, subjected to heat stresses or under the treatment of MeJA and abscisic acid (ABA), were determined. Consequently, this study will provide a theoretical basis for further study of the biological role of JAZ proteins in *C. lacryma-jobi*.

## 2. Results

### 2.1. Systematic Identification of Jasmonate ZIM-Domain Family Members in C. lacryma-jobi

An HMM search was carried out on the *C. lacryma-jobi* genome and a total of 20 *JAZ* genes were characterized and defined as *ClJAZ1*-*ClJAZ20* based on their physical position on the 10 chromosomes. As indicated in Table 1, the lengths of the 20 ClJAZ proteins ranged from 159 aa (ClJAZ6) to 409 aa (ClJAZ18), and the corresponding molecular masses ranged from 16.55 kD to 43.00 kD. The theoretical isoelectric points (pI) varied from 4.64 (ClJAZ11) to 11.18 (ClJAZ7), with an average pI of 8.60. The instability index (II) values ranged from 38.33 (ClJAZ4) to 84.60 (ClJAZ1), whereas aliphatic index (AI) values ranged from 58.78 (ClJAZ14) to 80.13 (ClJAZ6). The grand averages of hydropathicity (GRAVY) of ClJAZ1-ClJAZ20 varied from −0.679 (ClJAZ11) to –0.160 (ClJAZ3), suggesting that all ClJAZ proteins were hydrophilic. Subcellular localization showed that ClJAZ1-ClJAZ20 were targeted to the nucleus.

### 2.2. Phylogenetic Analysis of ClJAZs and Other Plant Jasmonate ZIM-Domain Proteins

**Figure 1:**
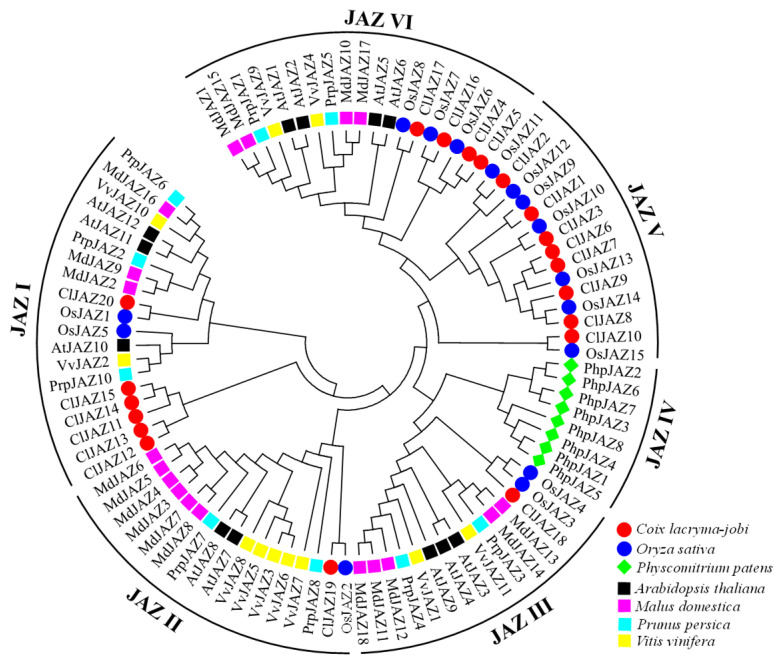
Phylogenetic tree of JAZ family proteins in *Arabidopsis thaliana*, *Coix lacryma-jobi*, *Malus domestica*, *Oryza sativa*, *Physcomitrium patens*, *Prunus persica* and *Vitis vinifera*.

To investigate the genetic distances between JAZ proteins, 93 JAZ proteins from *A. thaliana*, *C. lacryma-jobi*, *Malus domestica*, *O. sativa*, *Physcomitrium patens*, *Prunus persica*, and *Vitis vinifera* were recruited to establish an evolutionary tree (Figure 1). These 93 JAZ proteins were categorized into six subfamilies (I–VI), with 17, 8, 20, 14, 15, and 19 members, respectively. In *C. lacryma-jobi*, group V had the highest number of members (8), followed by groups III (6) and VI (4). Meanwhile, both groups I and IV harbored 1 member, *ClJAZ19* and *ClJAZ20*, respectively. Group II only contained bryophyte *P. patens*, while group V only included monocot plants *O. sativa* and *C. lacryma-jobi*. Notably, JAZ proteins between monocot and dicot plants exhibited a certain limit in the evolutionary tree, that is, JAZ proteins in dicot or monocot plants embedded into the same branch within each subgroup, suggesting that JAZ family proteins evolved in relatively independent directions based on the actual needs of monocot or dicot plants in terms of coping with abiotic and biotic stresses.

### 2.3. Chromosome Localization and Collinearity Analysis of ClJAZs Members

**Figure 2:**
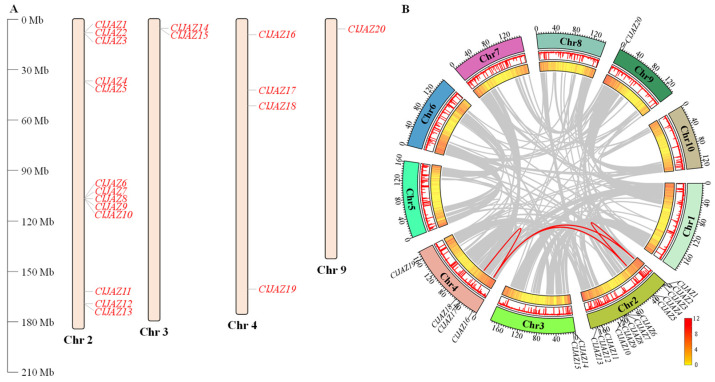
Chromosomal localization (**A**) and replication events (**B**) of *ClJAZ* family genes in *Coix lacryma-jobi*.

On the basis of the chromosome-level *C. lacryma-jobi* genome, *ClJAZ1*-*ClJAZ20* were unevenly distributed across 4 chromosomes, including Chr 2, Chr 3, Chr 4, and Chr 9, which mapped 13, 2, 4 and 1 ClJAZ proteins (Figure 2A), respectively. Furthermore, 5 colonies (*ClJAZ1*-*ClJAZ3*, *ClJAZ4*-*ClJAZ5*, *ClJAZ6*-*ClJAZ10*, *ClJAZ12*-*ClJAZ13*, and *ClJAZ14*-*ClJAZ15*) were located on Chr 2 and Chr 3. To better understand the intra-species collinearity of the 20 ClJAZ genes, a total of four gene duplication events (*ClJAZ2* and *ClJAZ5*, *ClJAZ2* and *ClJAZ16*, *ClJAZ5* and *ClJAZ16*, and *ClJAZ16* and *ClJAZ17*) took place on Chr 2 and Chr 4 (Figure 2B).

**Figure 3:**
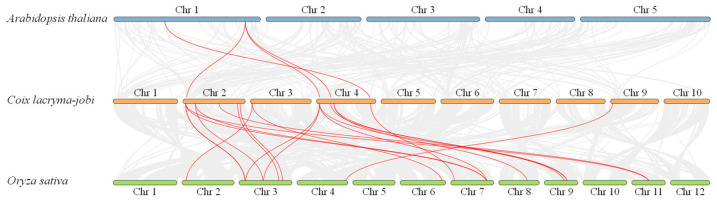
Collinearity analysis of *JAZ* genes in *Coix lacryma-jobi*, *Arabidopsis thaliana* and *Oryza sativa*.

Simultaneously, to further understand the inter-species collinearity of JAZ proteins among *C. lacryma-jobi*, *A. thaliana*, and *O. sativa*, 4 and 16 conserved syntenic regions in *C. lacryma-jobi* were mapped into the genomes of the model plants *A. thaliana* and *O. sativa* (Figure 3), respectively. The results suggested that the *JAZ* genes were more highly conserved in monocots than dicots.

### 2.4. Conserved Motifs and Gene Structure of ClJAZs Members

**Figure 4:**
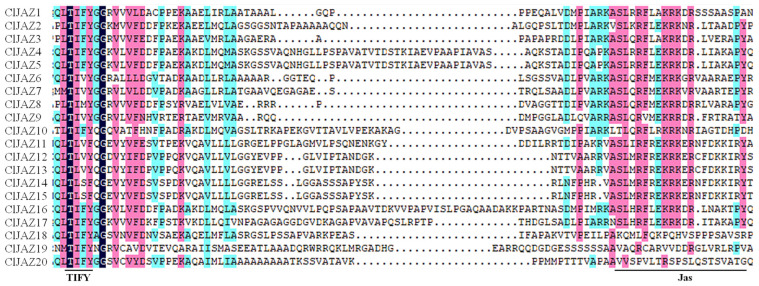
Multiple sequences alignment of 20 ClJAZ proteins. TIFY: TIFY domain. Jas: Jas domain.

To characterize the C. *lacryma-jobi* Jasmonate ZIM-domain members, a multiple-sequence alignment of 20 ClJAZ proteins was performed by DNAMAN v. 9.0 software (Figure 4), showing that the 20 ClJAZ proteins contained two typical domains, namely TIFY and Jas.

**Figure 5:**
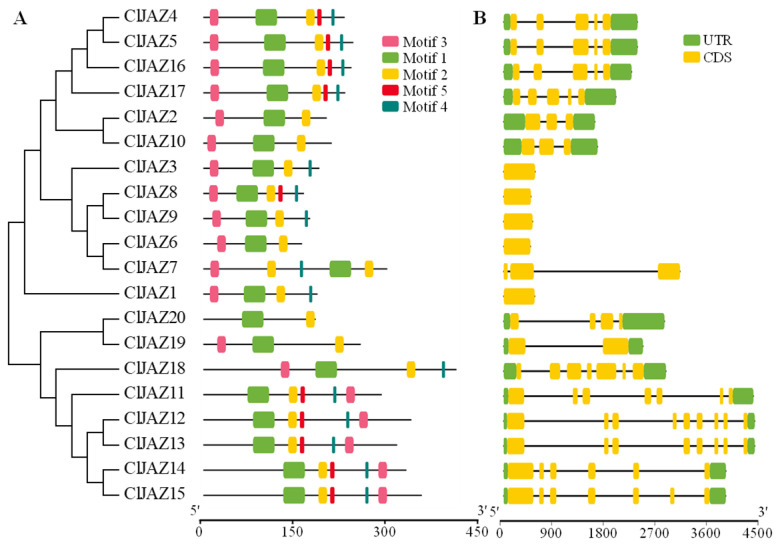
Conserved motifs (**A**) and gene structure (**B**) of ClJAZ members in *Coix lacryma-jobi*.

To further identify the characteristics of ClJAZ proteins, conserved motifs were predicted using the MEME online suite, and five conserved motifs were analyzed (Figure 5A, Table S1). ClJAZ1-ClJAZ20 contained two to five conserved motifs; however, all ClJAZ proteins harbored motif 1 and motif 2, suggesting that they were fundamental elements in the plant JAZ family.

To gain insights into the diversification of ClJAZ members, exon-intron organization among ClJAZ1-ClJAZ20 were compared (Figure 5B). Notably, six ClJAZ members (*ClJAZ1*, *ClJAZ3*, *ClJAZ6*, *ClJAZ7*, *ClJAZ8*, and *ClJAZ9*) only possessed 1–3 coding sequences, the remaining 14 members presented two untranslated regions and 2–9 coding sequences. Overall, 20 *ClJAZ* genes exhibited variable organization; however, *ClJAZ* genes in the same branch shared a similar gene structure.

### 2.5. Cis-Regulating Elements Analysis of the Promoter Sequences Before ClJAZs Genes

**Figure 6:**
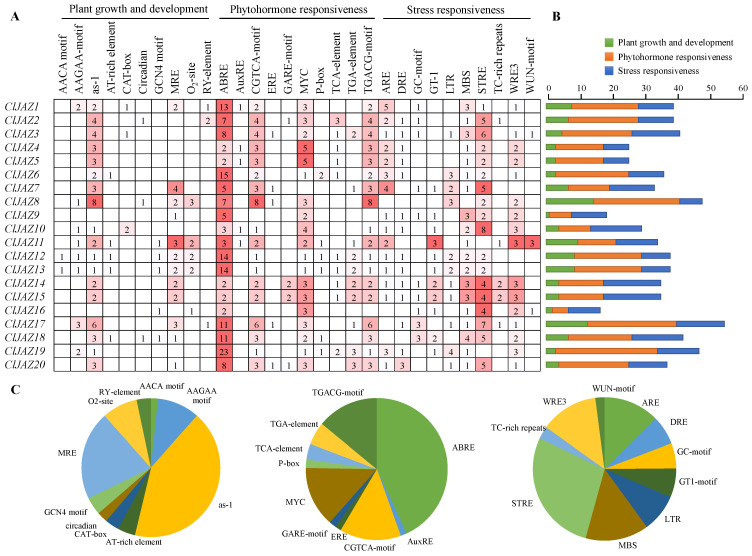
Cis-regulating element analysis of *ClJAZ* genes in *Coix lacryma-jobi*. (**A**) Heatmap of three biological categories for 730 *cis*-acting elements. (**B**) Number of *cis*-acting elements in each *ClJAZ* gene. (**C**) Histogram of different *cis*-acting elements in each category.

Cis-regulating elements (CREs) play an important role in the regulation of gene expression. The upstream 2000 bp of 20 *ClJAZs* genes were submitted into the PlantCARE database, resulting in 30 kinds of CREs, which were classified according to their biological roles in plant growth and development, phytohormone responsiveness, and stress responsiveness, with 121, 364, and 245 CREs in each category, respectively (Figure 6). The promoter sequences of *ClJAZ1*-*ClJAZ20* contained 10–56 CREs, with the least number in *ClJAZ16* and the largest number in *ClJAZ17*. Activation sequence-1 (as-1), the ABA responsive element (ABRE), and the stress response element (STRE) occupied the largest proportion in plant growth and development, phytohormone responsiveness, and stress responsiveness, with 51, 157, and 68 CREs in each category, respectively. Intriguingly, ABRE accounted for the biggest portion of the elements (21.51%), suggesting that *ClJAZ* family genes were likely to be induced by ABA treatment. Three kinds of CREs, including CGTCA-motif, MYC, and TGACG-motif, were abundantly present and were stimulated by MeJA. Moreover, *ClJAZ* family genes had numerous CREs in response to light, low temperature, drought, injury, and salicylic acid, indicating that *ClJAZ* genes played essential roles in the growth and development, hormone responses, and stress responses of *C. lacryma-jobi*.

### 2.6. Expression Patterns of ClJAZs Genes in Different Tissues

**Figure 7:**
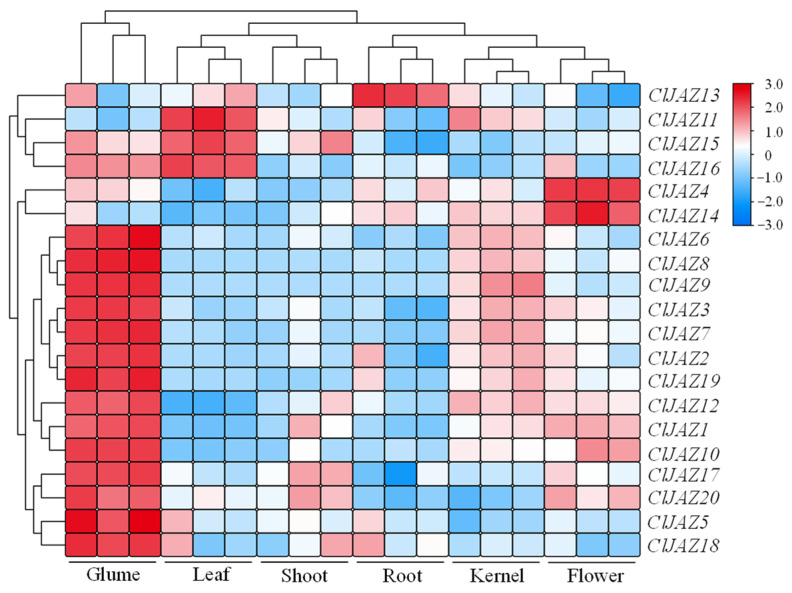
Expression profiles of 20 *ClJAZ* genes in different tissues of *Coix lacryma-jobi*.

To determine the expression dynamics of *ClJAZ1*-*ClJAZ20* across different tissues, qRT-PCR assays were carried out and varieties in expression were represented in the form of heatmaps (Figure 7). *ClJAZ1*-*ClJAZ20* were differently expressed in six tissues, including the glumes, leaves, shoots, kernels, flowers, and roots of *C. lacryma-jobi*. The majority of ClJAZ genes (70%) were highly expressed in the glumes, *ClJAZ11*, *ClJAZ15*, and *ClJAZ16* were mainly expressed in the leaves, *ClJAZ4* and *ClJAZ14* were highly expressed in flowers, and *ClJAZ13* was mainly expressed in the roots, suggesting that these genes might play a vital role in the growth and development of *C. lacryma-jobi* plants.

### 2.7. Transcriptome Analysis of C. lacryma-jobi Under Heat Stress

**Figure 8:**
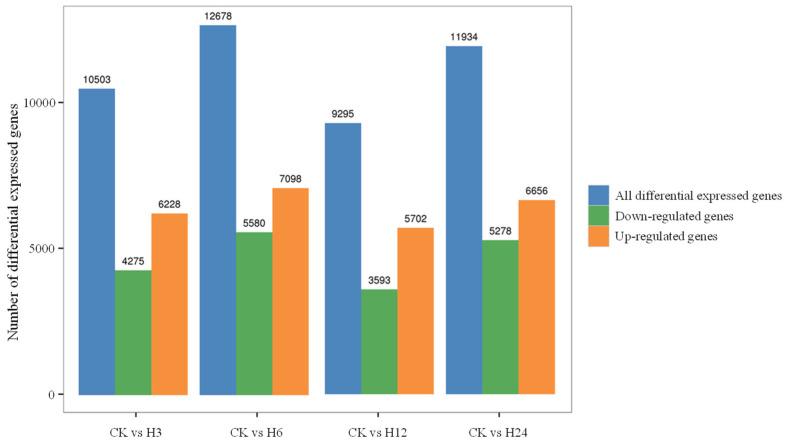
Differentially expressed genes subject to control and heat stress. CK, control. H3, H6, H12, and H24 indicate heat stress for 3, 6, 12, and 24 h, respectively.

To investigate transcriptional regulation under heat stress, *C. lacryma-jobi* was treated at 42℃ for 0, 3, 6, 12, and 24 h for RNA sequencing. A total of 175.23 Gb of clean data were acquired, containing an average of 38,940,475 clean reads with Q30 values ranging from 91.78% to 93.74% (Table S2). Differentially expressed genes (DEGs) were identified at five time points. Compared with the control group, heat stress at 3 h resulted in 10,503 DEGs with 6228 up-regulated genes and 4275 down-regulated genes. Similarly, 12,678 DEGs were found at 6 h with 7098 up-regulated genes and 5580 down-regulated genes, 9295 DEGs were found at 12 h with 5702 up-regulated genes and 3593 down-regulated genes, and 11,934 DEGs were acquired at 24 h with 6656 up-regulated genes and 5278 down-regulated genes (Figure 8 and Figure S1).

**Figure 9:**
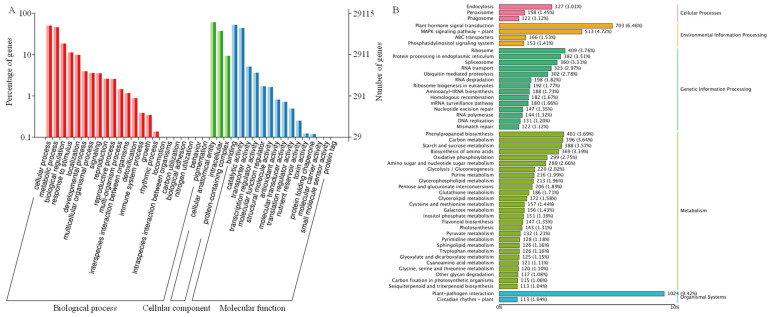
GO (**A**) and KEGG (**B**) enrichment analysis of differential expressed genes under control and heat stress.

To reveal the role of all picked DEGs, GO enrichment analysis was implemented (Figure 9A). A total of 29,115 DEGs were annotated and classified into three categories (biological process, cellular component, and molecular function). In terms of biological process, cellular process and metabolic process ranked in the top two, while binding and catalytic activity ranked in the top two in terms of molecular function. In addition, KEGG enrichment analysis showed that DEGs were mainly concentrated in cellular processes, environmental information processing, genetic information processing, the metabolism, and organismal systems (Figure 9B). Plant hormone signal transduction was mostly enriched in environmental information processing, and phenylpropanoid biosynthesis accounted for the largest proportion of activity in terms of metabolism. It suggested that *C. lacryma-jobi*’s behavior under heat stress may be closely related to hormones and the phenylpropanoid metabolism. For the genes *ClJAZ1*-*ClJAZ20*, transcription factor activity was the most enriched in terms of molecular function, while biological regulation and metabolic process were mainly enriched in terms of biological processes (Figure S2).

### 2.8. Expression Patterns of ClJAZs Genes Subjected to Heat Stress

**Figure 10:**
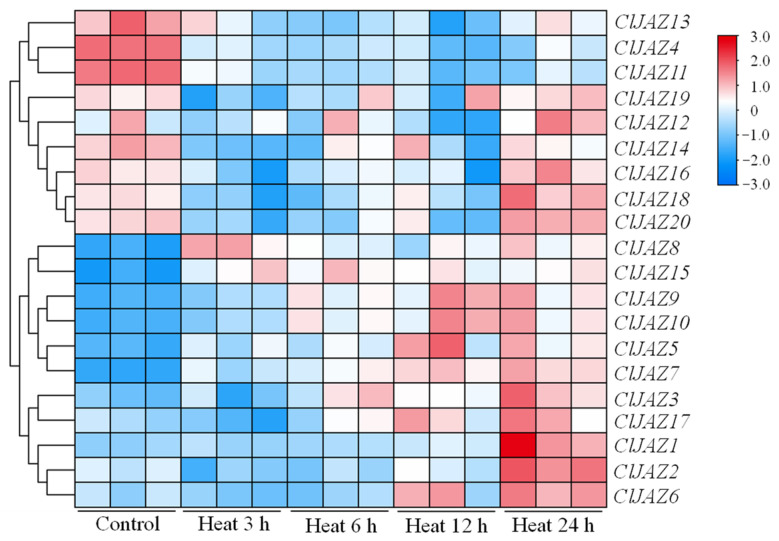
Expression profiles of 20 *ClJAZ* genes under heat stress for 0, 3, 6, 12, and 24 h.

To detect the expression patterns of *ClJAZ1*-*ClJAZ20* under heat stress for 3, 6, 12, and 24 h, the genes’ dynamic expressions were observed using RNA-seq (Figure 10). The transcript levels of *ClJAZ1*-*ClJAZ20* were broadly split into two groups: high and low expression in control treatment ranging from 2.92 to 254.10. *ClJAZ1*, *ClJAZ2*, *ClJAZ3*, *ClJAZ5*, *ClJAZ7*, *ClJAZ8*, *ClJAZ9*, *ClJAZ10*, *ClJAZ15*, and *ClJAZ17* showed relatively low expression in control treatment compared to their expression under heat stress. *ClJAZ4*, *ClJAZ11*, *ClJAZ12*, and *ClJAZ13* were relatively more highly expressed in control treatment than during heat stress, but the expression levels of *ClJAZ14*, *ClJAZ16*, *ClJAZ18*, *ClJAZ19*, and *ClJAZ20* decreased first and then increased.

**Figure 11:**
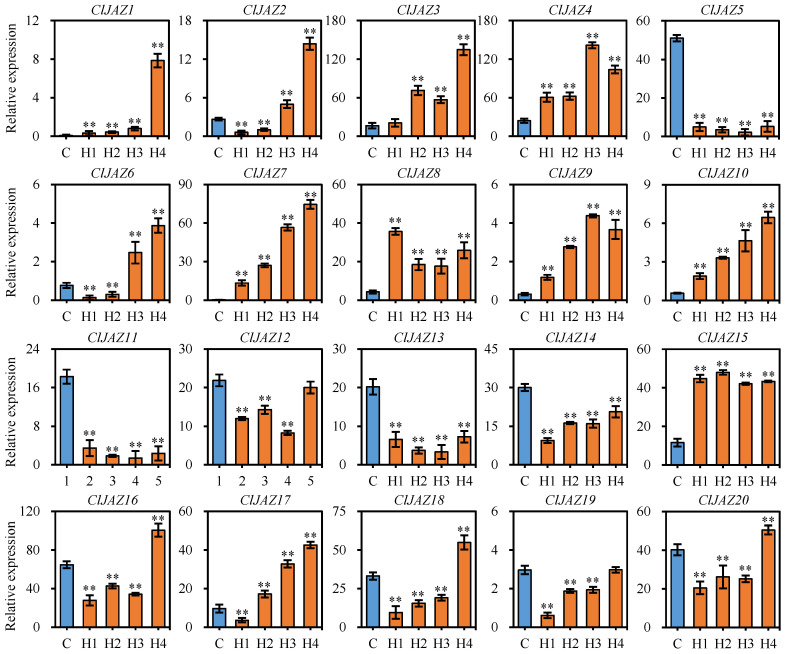
Expression profiles of 20 ClJAZ genes under heat stress in *Coix lacryma-jobi*. C, control. H1, H2, H3, and H4 are exposed to high temperature (42 °C) for 0, 3, 6, and 12 h, respectively. ** is indicated significant difference between C and H treatment at 0.01 level.

Based on the qRT-PCR results, *ClJAZ5*, *ClJAZ11*, *ClJAZ12*, *ClJAZ13*, *ClJAZ14*, and *ClJAZ19* were down-regulated, whereas *ClJAZ16*, *ClJAZ17*, *ClJAZ18*, and *ClJAZ20* were reduced first and then increased, and the other 10 *ClJAZ* genes were significantly up-regulated (Figure 11).

### 2.9. Expression Patterns of ClJAZs Genes Exposed to ABA and MeJA Treatment

**Figure 12:**
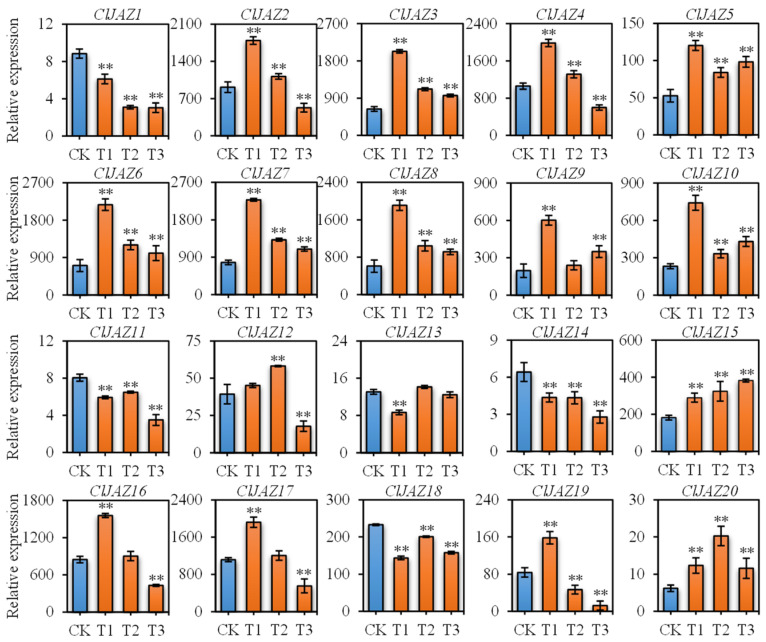
Expression profiles of 20 *ClJAZ* genes under ABA induction for 0, 24, 48 and 72 h. ** indicates a significant difference between C and H treatment at 0.01 level.

**Figure 13:**
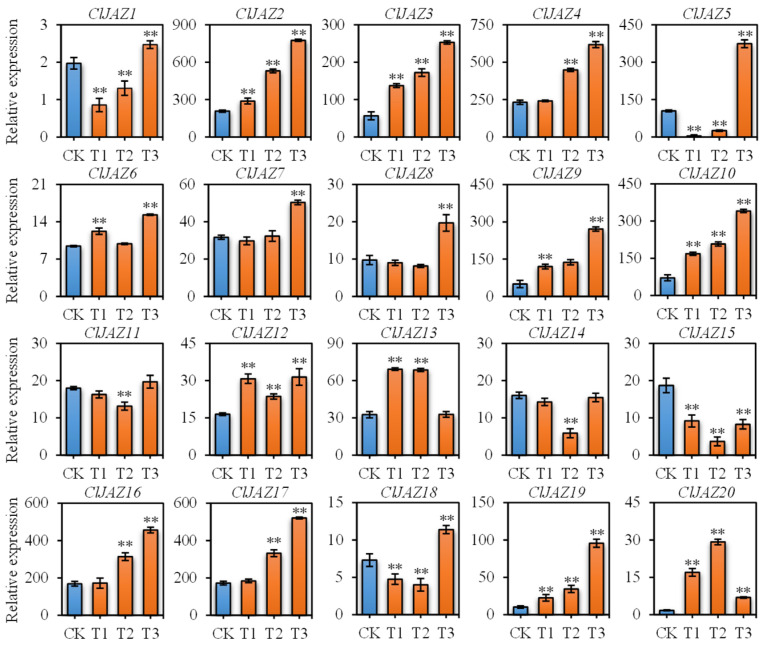
Expression profiles of 20 *ClJAZ* genes under MeJA induction for 0, 12, 24 and 48 h. ** is indicated significant difference between C and H treatment at 0.01 level.

Since abundant cis-regulating elements respond to ABA and MeJA stimuli (Figure 6), the expression patterns of *ClJAZ1*-*ClJAZ20* subjected to ABA (Figure 12) and MeJA (Figure 13) treatment were evaluated. Under ABA induction, *ClJAZ1*, *ClJAZ11*, *ClJAZ13*, *ClJAZ14*, and *ClJAZ18* were reduced 0.34–0.86-fold, while the other 15 *ClJAZ* genes showed 1.44–3.28-fold increases. Similarly, *ClJAZ11*, *ClJAZ14*, and *ClJAZ15* were reduced 0.04–0.73-fold when exposed to MeJA treatment, while the other 17 *ClJAZ* genes increased 1.43–17.24-fold. The results suggested that the *ClJAZ1*-*ClJAZ20* genes could be inducible under external ABA and MeJA.

## 3. Discussion

The Jasmonate ZIM-domain protein is a crucial member of plant-specific TF and has been identified in multiple monocots and dicots [5,8,9]. The JAZ protein represses the activity of DNA-binding TFs that modulate the transcript levels of JA-inducible genes [21]. Currently, the JAZ gene family is not only associated with different developmental processes, but also plays an essential role in the adaptability of medicinal plants to adverse conditions, including high temperatures, water deficits, and salinity [22,23]. The chromosome-level genome analysis of *C. lacryma-jobi* greatly furthers the investigation of the JAZ gene family through a genome-wide survey. Herein, a total of 20 ClJAZ family members were excavated and characterized in *C. lacryma-jobi* for the first time (Table 1; Figure 1). The amount of JAZ genes in *C. lacryma-jobi* was greater than the amount in *A. thaliana* (13) [8], *Ficus carica* (10) [24], *O. sativa* (15) [9], *Sorghum bicolor* (17) [25], and *Zea mays* (16) [26], and less than the amount in *Glycine max* (33) [27] and *S. spontaneum* (49) [13]. Additionally, the genome size of *C. lacryma-jobi* (1.73 G) is almost 13.12 times the size of *A. thaliana* (135 Mb) [28], 4.59 times the size of *O. sativa* (385.7 Mb) [29], 2.45 times the size of *S. bicolor* (722.96 Mb) [30], 0.81 times the size of of *Z. mays* (2178.6 Mb) [31], and one-sixth the size of *S. spontaneum* (10.4 Gb) [32]. Hence, the amount of JAZ family members in different plants may not be proportional to their genome size. According to the phylogenetic tree (Figure 1), a close relationship was shown between the monocot *O. sativa* and the dicot *A. thaliana*. It is inferred that duplication and genome evolution are indispensable in enlarging the numbers of the JAZ gene family [8]. In addition, *ClJAZ1-ClJAZ20* were randomly targeted to four chromosomes, and exhibited high collinearity with *O. sativa* than *A. thaliana* (Figure 2 and Figure 3), which might result from the differential accumulation of specialized metabolites [33]. According to GO and KEGG enrichment, 20 *ClJAZ* genes were involved in the biological and metabolic processes, suggesting that they were indispensable for *C. lacryma-jobi*’s developmental processes (Figure 9).

The ClJAZ members were diverse in size, Mw, pI, II, and AI, but demonstrated similar GRAVYs and localizations, which were in line with those found in *S. lycopersicum* [10]. The 20 ClJAZ proteins contained conserved motif 1 and 2 (Figure 5), consisting of TIFY and Jas domains (Figure 4), which were consistent with those in previous reports [15,17].

CREs are situated before 20 *ClJAZ* genes and are conductive to predicting their potential functions in response to abiotic stresses [34,35]. Genes *ClJAZ1-ClJAZ20* contained 730 CREs, mostly consisting of phytohormone-responded CREs, which were similar to those in *C. annuum* [12], with the highest amount of CREs responding to ABRE, CGTCA-motif, MYC, and TGACG-motif, suggesting that most *ClJAZ* genes could be regulated by ABA and MeJA (Figure 6). As shown in Figure 12, 25% of the *ClJAZ* genes were down-regulated when subjected to ABA induction, whereas 75% of the *ClJAZ* genes were up-regulated. JAZ members are vital to JA signaling transduction within many plants, for instance, *A. thaliana*, *C. annuum*, and *O. sativa* [8,9,12]. ABRE can bind to upstream TF and stimulate the transcript levels of ABA-inducible genes, thus enhancing plant stress tolerance [36]. MeJA also plays a significant role in the adaption of plants to adverse stresses [2]. In the present study, 17 of 20 *ClJAZ* genes were up-regulated under MeJA treatment, but the other 3 *ClJAZ* genes displayed an opposite tendency (Figure 13). Transgenic *A. thaliana* seedlings overexpressing the *AtJAZ3* gene, which encodes a JAZ member that contains both the TIFY and Jas domains, leads to an MeJA-insensitive phenotype [8]. Moreover, multiple stress-related CREs, such as STRE and MBS, were also displayed in the promoter regions of genes *ClJAZ1-ClJAZ20*, inferring that they might be influenced by various hormones, thereby sustaining high stress resistance.

*C. lacryma-jobi* is an herbaceous crop and exhibits adaptability to abiotic stresses, such as drought, high temperature, and salinity [18]. Under high temperature, 9295–12,678 DEGs were identified (Figure 8), mainly resulting in plant hormone signal transduction and several metabolic processes. Using combined RNA-seq and qRT-PCR technology, genes *ClJAZ1-ClJAZ20* were significantly induced through exposure to heat stress (Figure 10 and Figure 11). Furthermore, the expression profiles of 58 flavonoid biosynthetic genes were determined, and most of them were reduced, or increased first and then reduced (Figure S3), indicating that heat stress may lead to a decrease in flavonoid content. Multiple *CsJAZ* genes showed tissue-specific expression profiles, for instance, preferential expression in photosynthetic organs (leaves; *ClJAZ11*, *ClJAZ15*, and *ClJAZ16*; Figure 7). Notably, 14 *ClJAZ* genes were highly expressed in glumes, which is an indispensable outermost part of *C. lacryma-jobi* florets [18,20], and which provides the mechanical protection for the pharmaceutically active kernels. These *ClJAZ* genes may affect the JA signal pathway and modulate glume development during the impregnation of *C. lacryma-jobi*. In *Camellia sinensis*, CsJAZ6 directly interacts with downstream genes, such as *CsEGL3* and *CsTTG1*, thereby reducing catechin accumulation during exposure to high temperatures [37]. In sum, *ClJAZ15* was down-regulated when exposed to MeJA treatment, but significantly up-regulated under ABA treatment and under heat stress, suggesting that *ClJAZ15* may be repressing flavonoid production, and thereby involved in ABA-mediated heat adaptation to high-temperature stress. These findings are generally consistent with the previous results [12], inferring that JAZ members act in response to heat stress, which provides new information and resources for the further functional characterization of JAZ proteins in *C. lacryma-jobi*.

## 4. Materials and Methods

### 4.1. Genome-Wide Identification of Jasmonate ZIM-Domain Family Members in C. lacryma-jobi

The annotation and chromosome-level genome of *C. lacryma-jobi* were downloaded from the National Genomics Data Center (https://ngdc.cncb.ac.cn/, GWHAAYR00000000; accessed on 28 May 2024). The 12 JAZ family proteins in *A. thaliana* were recruited from the TAIR database (www.arabidopsis.org, accessed on 6 June 2024) and were used to search ortholog sequences against the high-quality *C. lacryma-jobi* genome by the Tbtools v2.119 platform [38]. Sequences with higher homology and coverage and with lower expected value (E-value < 1 × e^−10^) were screened for further bioinformatic validation. Two Markov models (HMM) of the TIFY domain (PF06200) and the JAS domain (PF09425) were acquired from the PFAM database (http://pfam.xfam.org/, accessed on 6 June 2024) and we employed JAZ members for target hits within the TIFY and JAS domains through HMMER v 3.4 software (http://hmmer.org/, accessed on 6 June 2024). The candidate JAZ family members were sequentially merged and submitted to NCBI CD-Search (www.ncbi.nlm.nih.gov/cdd, accessed on 6 June 2024) through the above two protocols to verify these conserved domains.

The physicochemical properties, including molecular weight (Mw), isoelectric point (pI), instability index (II), aliphatic index (AI), and grand average of hydropathicity (GRAVY), were in-line analyzed through the ExPASy server (www.expasy.org, accessed on 6 June 2024). The subcellular localization of ClJAZ1-ClJAZ20 was predicted through the Plant-mPLoc server (www.csbio.sjtu.edu.cn/bioinf/plant-multi/, accessed on 26 July 2024) and the WoLF PSORT tool (https://wolfpsort.hgc.jp, accessed on 26 July 2024).

### 4.2. Phylogenetic Analysis of ClJAZ Family Proteins

To understand the evolutionary relationship between different species, the JAZ proteins from monocots (*O. sativa* and *C. lacryma-jobi*) and dicots (*A. thaliana*, *M. domestica*, *Prunus persica* and *V. vinifera*), as well as a bryophyte (*Physcomitrium patens*), were acquired from Phytozome v13 (https://phytozome.jgi.doe.gov, accessed on 6 June 2024). These 93 JAZ proteins (Table S3) were sequence-to-sequence aligned with ClustalX v2.0 (www.clustal.org, accessed on 26 July 2024) under the default settings. The evolutionary tree was constructed via the neighbor-joining method through MEGA v11.0 (www.megasoftware.net, accessed on 2 November 2024) with 1000 bootstrap repetitions.

### 4.3. Conserved Motifs, Gene Structure and Protein–Protein Interaction of ClJAZ Members

The conserved motifs of ClJAZ proteins were identified through the MEME server (https://meme-suite.org/, accessed on 28 July 2024), and the maximum number of motifs was identified as 5, while the amino acid residues ranged from 6 to 50. The gene structure of *ClJAZ* genes was visualized with the Tbtools v2.119 platform. The STRING v12 server (https://string-db.org, accessed on 28 July 2024) was utilized to generate a protein–protein interaction network.

### 4.4. Chromosomal Location and Collinearity Analysis of ClJAZ Genes

According to the annotation of the high-quality *C. lacryma-jobi* genome, the chromosomal location of 20 *ClJAZ* genes was generated with the Tbtools v2.119 platform [38].

Collinearity analysis among *C. lacryma-jobi*, *A. thaliana*, and *O. sativa* was carried out through one-step MCScanX module in the Tbtools v2.119 platform [38].

### 4.5. Analysis of Cis-Regulating Elements upon the ClJAZ Promoters

The 2000 bp upstream regions from the initiation codon (ATG) of *ClJAZ* genes were excavated from the high-quality *C. lacryma-jobi* genome, and were uploaded to the PlantCARE server (http://bioinformatics.psb.ugent.be/, accessed on 28 July 2024). The obtained results were analyzed and visualized according to their biological functions.

### 4.6. Transcriptome Analysis of ClJAZ Gene Expression in Different Tissues

The expression patterns of *ClJAZ1-ClJAZ20* in six different tissues, including the glumes, leaves, shoots, kernels, flowers, and roots of *C. lacryma-jobi*, were investigated. RNA-seq reads were downloaded from NCBI under the BioProject accession of PRJNA544168. All clean reads were annotated and aligned to the high-quality *C. lacryma-jobi* genome using the HISAT2 program (https://github.com/DaehwanKimLab/hisat2, accessed on 28 July 2024). The StringTie tool (https://github.com/gpertea/stringtie, accessed on 2 August 2024) was subsequently utilized to calculate the FPKM values. Afterwards, the log2-transformed FPKM values were used to generate a heatmap through the Tbtools v2.119 platform [38].

### 4.7. Transcriptome Analysis of ClJAZ Gene Expression Under Heat Stress

To understand the effects of exposure to heat stress, *C. lacryma-jobi* plants were cultivated at 42 °C for 0, 3, 6, 12, and 24 h, and transcriptomically analyzed via RNA-seq against the BMKCloud platform (https://www.biocloud.net/, accessed on 5 August 2024). Raw reads with high unknown base N content (greater than 5%), linker contamination, and low quality (quality value less than 15) were eliminated through SOAPnuke (https://github.com/BGI-flexlab/SOAPnuke, accessed on 5 August 2024). Gene expression levels were calculated according to FRKM values for genes in the control environment and those under heat stress. The acquired unigenes were evaluated via DESeq2 software (https://github.com/thelovelab/DESeq2, accessed on 5 August 2024) for DEGs. Four protein databases, including gene ontology (GO), NCBI non-redundant protein sequences (NR), the Kyoto encyclopedia of genes and genomes (KEGG), and the plant resistance genes database (PRGdb), were co-employed for transcriptome annotation. All FPKM values of each treatment were converted to log2 and used to generate a heatmap using Tbtools v2.119.

### 4.8. Plant Growth and Experimental Treatments of C. lacryma-jobi

*C. lacryma-jobi* ‘Zheyi no 2′ (a high-stress resistance; genetic breeding by Prof. Xiaoxia Shen at Zhejiang Chinese Medical University, China; accession number 2014004; website: https://www.zjitcm.com/info/details/7/615.html, accessed on 5 August 2024) were cultivated in a greenhouse at 25 °C under a light/dark cycle of 14/10 h. The three-month-old plantlets were treated with PEG 6000 simulated drought (10%, *w*/*v*), heat treatment (42 °C), and hormone induction (50 μM MeJA and 100 μM ABA), respectively. Ultimately, the samples were immersed in liquid nitrogen and stored at -80 °C. Three independent replicates were implemented.

### 4.9. RNA Extraction and Quantitative Real-Time PCR Analysis

Total RNA was isolated through the Quick RNA Isolation Kit (Huayueyang Biotechnology Co., Beijing, China) following the supplier’s instructions, and was reverse transcribed to cDNA through the PrimeScript Reagent Kit with gDNA Eraser (Takara, Dalian, China). Quantitative real-time PCR was implemented through 2× iTaq™ Universal SYBR^®^ Green Supermix (Bio-Rad Laboratories, Hercules, CA, USA) on a StepOnePlus™ Real-Time PCR System (Applied Biosystems, Waltham, MA, USA). The PCR reactive system and program complied with the previous protocol [39]. The relative expression was estimated in accordance with the 2^−∆∆Ct^ algorithm. *ClEF-1α* (Table S4) was employed as a house-keeping gene [40].

## 5. Conclusions

To summarize, 29,115 DEGs were annotated and enriched in hormone transduction, environmental stimulus, and phenylpropanoid metabolism under control and heat stress for 3, 6, 12, and 24 h. With the release of a high-quality *C. lacryma-jobi* genome, 20 ClJAZ protein sequences were systematically characterized. Tandem and segmental duplication might facilitate the expansion of the ClJAZ family. Genes *ClJAZ1-ClJAZ20* displayed stronger multi-variable collinearity with monocots than dicots. In terms of the integration of promoter elements and expression profiles, *ClJAZ1*-*ClJAZ20* were tissue-specific and exhibited various responses to many stressors, such as ABA, heat, and MeJA. Intriguingly, MeJA-inhibited *ClJAZ15* was highly expressed in the leaves and responded to ABA-inducible high temperature stress, meaning that it might be a critical heat-stress regulator. Taken together, the current study provides a foundation for further characterization of *ClJAZ* genes in *C. lacryma-jobi*.

## Figures and Tables

**Table 1:** Physicochemical properties of ClJAZ family members in *Coix lacryma-jobi*.

Name	Size/aa	Mw/kD ^1^	pI ^2^	II ^3^	AI ^4^	GRAVY ^5^	Localization ^6^
ClJAZ1	184	19.17	9.36	84.60	70.87	−0.234	Nucleus
ClJAZ2	199	21.18	9.77	66.41	68.44	−0.424	Nucleus
ClJAZ3	187	19.41	7.83	61.91	74.06	−0.160	Nucleus
ClJAZ4	228	24.00	6.62	38.33	69.61	−0.368	Nucleus
ClJAZ5	242	25.51	6.62	41.85	75.66	−0.287	Nucleus
ClJAZ6	159	16.55	10.09	45.17	80.13	−0.272	Nucleus
ClJAZ7	297	31.27	11.18	52.52	68.96	−0.468	Nucleus
ClJAZ8	162	17.39	9.84	71.82	76.48	−0.244	Nucleus
ClJAZ9	172	18.53	10.47	67.92	75.00	−0.257	Nucleus
ClJAZ10	207	22.28	10.05	46.08	68.02	−0.416	Nucleus
ClJAZ11	288	30.92	4.64	62.42	64.83	−0.679	Nucleus
ClJAZ12	336	35.90	8.99	51.68	66.31	−0.494	Nucleus
ClJAZ13	313	33.45	8.94	54.62	65.88	−0.489	Nucleus
ClJAZ14	328	35.01	5.11	53.43	58.78	−0.628	Nucleus
ClJAZ15	353	37.57	5.12	55.84	64.28	−0.565	Nucleus
ClJAZ16	239	25.35	9.25	47.20	74.85	−0.426	Nucleus
ClJAZ17	229	23.84	9.28	43.83	74.76	−0.403	Nucleus
ClJAZ18	409	43.00	9.83	70.23	67.97	−0.281	Nucleus
ClJAZ19	254	25.55	9.78	64.91	69.76	−0.350	Nucleus
ClJAZ20	181	19.22	9.26	77.91	77.79	−0.367	Nucleus

^1^ Mw, molecular weight. ^2^ pI, isoelectric point. ^3^ II, instability index. ^4^ AI, aliphatic index. ^5^ GRAVY, grand average of hydropathicity. ^6^ Localization were predicted by Plant-mLOC and WoLF server.

## Data Availability

The chromosomal-scale genome data of *Coix lacryma-jobi* are available at the NCBI under the BioProject of PRJCA001469. RNA-seq data of *C. lacryma-jobi* at different tissues and heat stress can be obtained from NCBI under the BioProject accession of PRJNA544168 and PRJNA812268, respectively. All protocols were carried out in accordance with relevant guidelines and regulations. All experimental studies on plants complied with relevant institutional, national, and international guidelines and legislation.

## Supplementary Materials

The following supporting information can be downloaded at: https://www.mdpi.com/article/10.3390/plants13223230/s1, Figure S1 The cultivated *Coix lacryma-jobi* in seedling nursery of Hangzhou, China; Figure S2 Venn diagram of differential expressed genes under control and heat stress. H3, H6, H12, H24 indicates heat stress for 3, 6, 12, 24 h, respectively. CK, control; Figure S3 GO enrichment analysis of *ClJAZ1-ClJAZ20* genes in *Coix lacryma-jobi*; Figure S4 Expression profiles of flavonoid biosynthetic genes under heat stress. Table S1 The information of motif 1–5 using MEME server; Table S2 Transcriptome information for control and heat stress. Table S3 The protein sequences of ClJAZ family in *Coix lacryma-jobi*. Table S4 Primers used for quantitative real-time PCR.
