# Supplementary material for: Systematic Survey and Analysis Reveal Jasmonate ZIM-Domain Gene Family in Coix lacryma-jobi Under High Temperature
Source: Plants (Basel). 2024 Nov 17;13(22):3230. doi: 10.3390/plants13223230 (PMC11598564; doi:10.3390/plants13223230)
Supplement: Supplementary file 1 [file plants-13-03230-s001.zip › plants-3274560-supplementary.pdf]

Supplementary Materials

Table legends and figure legends

Table S1 The information of motif 1-5 using MEME server.
Table S2 Transcriptome information for control and heat stress.
Table S3 The protein sequences of ClJAZ family in *Coix lacryma-jobi*.
Table S4 Primers used for quantitative real-time PCR.

Figure S1 The cultivated *Coix lacryma-jobi* in seedling nursery of Hangzhou, China.
Figure S2 Venn diagram of differential expressed genes under control and heat stress. H3, H6, H12, H24 indicates heat stress for 3, 6, 12, 24 h, respectively. CK, control.
Figure S3 GO enrichment analysis of *ClJAZ1-ClJAZ20* genes in *Coix lacryma-jobi*.
Figure S4 Expression profiles of flavonoid biosynthetic genes under heat stress. C, control. H3, H6, H12, and H24 indicates *Coix lacryma-jobi* plants exposed to heat stress for 3, 6, 12, and 24 h.

Table S1 The information of motif 1-5 using MEME server.

	Name	E-value	Sites	Width
Motif 1	AAAAQLTIFYGGRVLVFDDVPPEKAQELMLLAGGAE	$1.00 \times e^{-241}$	20	36
Motif 2	RKASLQRFLEKRKDR	$1.60 \times e^{-148}$	20	15
Motif 3	RRFAVACGLLSQYVR	$6.10 \times e^{-69}$	20	15
Motif 4	WLGLGT	$8.80 \times e^{-14}$	20	6
Motif 5	KAPYQVRK	$2.70 \times e^{-5}$	20	8

Table S2 Transcriptome information for control and heat stress.

Samples	Description	Clean reads	Clean bases	GC content	Q30
C1	Control	34 155 106	10 246 531 800	57.47%	93.09%
C2	Control	32 680 328	9 804 098 400	55.63%	93.49%
C3	Control	43 752 001	13 125 600 300	56.78%	92.71%
H31	42 °C for 3 h	7 936 194	2 380 858 200	59.32%	93.06%
H32	42 °C for 3 h	39 978 125	11 993 437 500	58.73%	93.30%
H33	42 °C for 3 h	46 798 603	14 039 580 900	58.42%	93.24%
H61	42 °C for 6 h	43 987 203	13 196 160 900	56.95%	93.74%
H62	42 °C for 6 h	40 641 603	12 192 480 900	57.02%	93.64%
H63	42 °C for 6 h	47 409 260	14 222 778 000	57.12%	92.89%
H121	42 °C for 12 h	40 972 208	12 291 662 400	56.72%	92.95%
H122	42 °C for 12 h	42 654 605	12 796 381 500	56.21%	93.03%
H123	42 °C for 12 h	42 925 130	12 877 539 000	56.80%	91.78%
H241	42 °C for 24 h	42 199 378	12 659 813 400	55.38%	92.74%
H242	42 °C for 24 h	36 945 923	11 083 776 900	55.07%	93.12%
H243	42 °C for 24 h	41 071 460	12 321 438 000	55.05%	92.54%

Table S3 The protein sequences of ClJAZ family in *Coix lacryma-jobi*.

Name	Protein sequences
ClJAZ1	MAAMPTDSITRRFAVACGVLSQYVRNGSAPAMTSPPPPPFLKQAPPPAMAG PAAAAAAQEMLSSGAAPQQLTIFYGGRVVVLDACPPEKAAELIRLAATAAA LGQPPPEQALVDMPIARKASLRRFLAKRKDRSSSAASPANDDRQDDDEPPV PKKGKMAAAATREDPSSSWLALGNLCSMHSR
ClJAZ2	MAAEQLQHQPPAKQAAAAGSRFAVTCGLLRQYMMEQGGSGGATRCLAPA VAMGLMPEADAAVAVQATTEERTTVLELFPQQAGTLKDEQRRKRKEPADG RAPLTIFYGGKMVVFDDFPKEKAEELMQLAGSGGSNTAPAAAAAQQNALG QPSLTDMPLARKVSLKRFLEKRKNRLTAADPYPAAARAGRVSRRPSSLPQ
ClJAZ3	MAMAAAEGKSRRFALACGVLSQYVKAEQQMAAAAAPAPRAPATTLSLMP GADVGAEAEQEPAAAARGEEMAGPTSMAAAAAAPPLTIFYGGRVVVFEDF PAEKAAEVMRLAAGAERAAPAPAPRDDLPIARKASLQRFLAKRKDRLVERA PYARPSPAEEAEKTKQQPASWLGLGGSTDAERLTIAL
ClJAZ4	MAPAKSGEKATSFAMACSLLSRYVRQNGAAAGDLGLAIRAEADANRTSAD TEKGETTKETMDLFPQNAGFGSEAAMMQAPDAREAEKRQLTIFYGGKVLV FDDFPAEKAKDLMQMASKGSSVAQNHGLLPSPAVATVTDSTKIAEVPAAPI AVASAQKSTADIPQAPKASLRRFLEKRKDRLIAKAPYQGSTSDATPVTKEMA EGQPWLGLGPQIANPDLSLCKEGSQ
ClJAZ5	MAPAKSGEKATSFAMACSLLSRYVRQNGAAAGDLGLAIRAEADANRTSAD TEKGETTKETMDLFPQNAGFGSEAAMMQAPDASWLPNLTAGNILILREAE KRQLTIFYGGKVLVFDDFPAEKAKDLMQMASKGSSVAQNHGLLPSPAVATV TDSTKIAEVPAAPIAVASAQKSTADIPQAPKASLRRFLEKRKDRLIAKAPYQG STSDATPVTKEMAEGQPWLGLGPQIANPDLSLCKEGSQ
ClJAZ6	MAAAAAAAPSGGTGNNTAATTTSQFAAACSALSQYVRAAEAERTRARPPV RPLPLMPGADVDQQEQPETAVQLTIVYGGRALLLDGVTADKAADLLRLAA AAARGGTEQPLSGSSVADLPVARKASLQRFMEKRKGRVAARAEPYRRPGDR RDHLTLAL
ClJAZ7	MATAGSVQGHGARFAAACDALSRTGTPSDAWRGPVRARGARCGGGAGA GAGNERAADHLVRRADGGARRRPADKAAQLLSGSRPRKARHGAASAGAK DDLPMARKMSLRQFMEKRKSRVAARALALQPSGRRRRGRGVAPGPSHSHA LTRRYHTAACYHCTTTTSYVKAAAEAERMHGTVPVRPLPLMPGADVDASP DGLDLAQAPAQMMTIVYGGRVLVLDDVPADKAAGLLRLATGAAVQEGAG AESTRQLSAADLPVARKASLQRFMEKRKVRVAARTEPYRRPDASDHLKLTL
ClJAZ8	MAPAGFSGRRQFDVACGVLSRCVKVQTATAGERAAAPTMLLMPGADVTP DVREEPLAPLTIMYGGRVVVFDDFPSYRVAELVLVAERRRPDVAGGTTDIPVA RKASLQRFMEKRRDRRLVARAPYGVRPAPAPASSKKRNQGEQEQGSWLGL GVPVPGGDAC
ClJAZ9	MAPFPKAEADCSSRRRFAVACSVLSQCVRAESAAAHSRSVSVVQAQAASPM LLMPGADVVSSEDTVPTPASAQLTIVYGGRVLVFNHVRTERTAEVMRVAAR QQDMPGGLADLQVARRASLQRVMEKRRDRFRTRATYAPAPAPARATAAGS VPKEQREKDTDRWLALGIPG
ClJAZ10	MAAPGYNRFAFTCARMRQFMTEQNRQVRMGDLVGSSSFQRPPLQLTPVPV ATGPAAAWETGAATLPLFPVATGTKIVRPEEAKATLTIFYQGQVATFHNFPA

DRAKDLMQVAGSLTRKAPEKGVTTAVLVPEKAKAGDVPSAAGVGMPPIAR
KLTLQRFLRKRKNRIAGTDHPDHNEDASPGKKRDSTGAGKNAAEDVPDD
ASWLRL

ClJAZ11 MPDTDVDTEMREAAAAAAAAPGGDDDSEGDTEEEEEEEDDEVDEDEEEEL
PAASPAPAEEQAAPAPISAMPGNPNQLTLVFQGEVYVFESVTPEKVQAVLLL
LGRGELPPGLAGMVLPSQNENKGYDDILRRTDIPAKRVASLIRFREKRKERN
FDKKIRYAVRKEVALRMQRRKGQFAGRASLEGESSAPGFDPGSQGSGLDFAS
RESKCQNCGTSEKMTPAMRRGPAGPRTLCNACGLMWANKGTLRSCPKAK
VESPVVAIEQGISDNKALVTPNNDNVAASNGEA

ClJAZ12 MCVSTITRARLSSILHTCAQTGACPRPGPSAHASPPSDPQPMAAEPAADDH
DPRPPLADGAAAAGGGDASSAAAVEALMSAASEQLTLVYQGDVYIFDPVP
PQKVQAVLLVLGGYEVPPGLVIPTANDGKNTTVAARRVASLMRFREKRKER
CFDKKIRYSVRKEVAQNCQGTNPGLPIASRHHLPLGTYPRMKRRKGQFAGR
SDFGDGASSSAACVSPANGEDDHFRETHCQNCGISSRLTPAMRRGPAGPRS
LCNACGLMWANKGTLRSPLNAPKMTQQHLVNPSKMGDTDDKNSIVLPVE
YNQATVKTDSMMPKQEQKLDIRPPTEEDIKAVS

ClJAZ13 MCVSTITRARLSSILHTCAQTGACPRPGPSAHASPPSDPQPMAAEPAADDH
DPRPPLADGAAAAGGGDASSAAAVEALMSAASEQLTLVYQGDVYIFDPVP
PQKVQAVLLVLGGYEVPPGLVIPTANDGKNTTVAARRVASLMRFREKRKER
CFDKKIRYSVRKEVAQKMKRRKGQFAGRSDFGDGASSSAACVSPANGEDD
HFRETHCQNCGISSRLTPAMRRGPAGPRSLCNACGLMWANKGTLRSPLNA
PKMTQQHLVNPSKMGDTDDKNSIVLPVEYNQATVKTDSMMPKQEQKLDIR
PPTEEDIKAVS

ClJAZ14 MSHHDGSKPYQPRRGPERHPQPVDGIAAPPPAAVAPSVEHLVAAAAEAEA
LNRFAAEPQMHEQEAGEEEEEDEEEDEMEEEEEDEQEGQHGGIGGEHVPM
DADAAAAAAAAAAGVQMDPHALVPGTVPPMATNQLTLSFQGEVYVFDSV
SPDKVQAVLLLLGGRELSSLGGASSSAPYSKRLNFPHRVASLMRFREKRKER
NFDKKIRYTVRKEVALRMQRNRGQFTSSKPKPDEIAASEMAAADGSPNWA
LVEGRPPSAAECHHCGTNATATPMMRRGPDGPRTLCNACGLMWANKHN
GSVMSAPGSELENAAVAMANGHESSSSGV

ClJAZ15 MSHHDGSKPYQPRRGPERHPQPVDGIAAPPPAAVAPSVEHLVAAAAEAEA
LNRFAAEPQMHEQEAGEEEEEDEEEDEMEEEEEDEQEGQHGGIGGEHVPM
DADAAAAAAAAAAGVQMDPHALVPGTVPPMATNQLTLSFQGEVYVFDSV
SPDKVQAVLLLLGGRELSSLGGASSSAPYSKRLNFPHRVASLMRFREKRKER
NFDKKIRYTVRKEVALRMQRNRGQFTSSKPKPDEIAASEMAAADGSPNWA
LVEGRPPSAAECHHCGTNATATPMMRRGPDGPRTLCNACGLMWANKGLL
RDLSKSPVPLQVIQSAPLLDGGHNGSVMSAPGSELENAAVAMANGHESSSS
GV

ClJAZ16 MAASARPGERATSFAVACSLLSRFVRQNGAAAAELGLGIKGEVEPQRTPATV
SLLPGAEGEEAEEAERRKETMELFPQSAGFGVKDAAAPREQENKDKPKQLT
IFYGGKVLVFDDFPADKAKDLMQLASKGSPVVQNVVLPQPSAPAAVTDKV
VPAPVISLPGAQAADAKKPARTNASDMPIMRKASLHRFLEKRKDRLNAKTP
YQTSPSDGAPVKKEPESQAWLGLGPNAVKSNLSLS

ClJAZ17 MAGRAPAARDKTSFAATCSLLSQYLKDKKGGLQGLGGLAMAPAAAESHIV

	AGAGAFRPPTTMNLLSALDAPPAEEPSEKATTGETKDRDKRTATGNPREAA<br>GDEAQQLTIFYGGKVVVFDKFPSTKVKDLLQIVNPAGAGAGGDGVDKAGA<br>PVAVAPQSLRPTPTHDGLSADLPIARRNSLHRFLEKRKDRITAKAPYQVNNS<br>SAGVEASKVEKPWLGLGQEATVKQEI
ClJAZ18	MERDFLAAIGKEQQQPHKEKAKAGAEESAYFGGAAAAPAMDWSFASKPG<br>AAPALMSFRSAAAREEPSFPQFSSSSSFEGAKHPAPRILTHQRSFGPDSAQYA<br>AVHRAQPQPPQHALNGARLIPVSSPFNHTNPMFRVQSSPSLPNGVAGAAA<br>ASFKQPPFAINNAVPSSTVGFYGTRDAVRPKTAQLTIFYAGSVNVFDNVSAE<br>KAQELMFLASRGSLPSSAPVARKPEASIFAPAKVTVPEILPAKQMLFQKPQH<br>VSPPPSAVSRPIPGILQAATLPRSASSSNLDSPVPKSSVPLAVPPVSQALTAQPA<br>TLATTTAAAIMPRAVPQARKASLARFLEKRKERVTTAAPYPSAKSPLESSDTF<br>GSVSANDKSSCTDIALSSNHDESLCLGQPRNISFSQESPSTKLQI
ClJAZ19	MEGGTGRDGGGVAGAAAAGQERRRRGAGCGGDEAESSDGSSGGGGGVEL<br>SLRLRTGAADDDDGAAAASAPPPPPPAAAVEARRNMTIFYNGRVCAVDVT<br>EVQARAIISMASEEATLAAADQRWRRQKLMRGADHGEARRQQDGDGESSS<br>SSSAAVAQRCARVVDDRGLVRLRPVAVAVAPPPSLAAAGVGVVGPPRQQPP<br>VVVEGIDPAAGLSMKRSLQLFLQKRKARTAAAVAPPYAGGGAGAGARQAQ<br>AVRR
ClJAZ20	MDLLERNIKKGEAEEEKARKQEEEAGERRNTQEPQQQQGQGLSLSLANGS<br>ARSAMLPMSNPSANPAQLTIFYGGSVCVYDSVPPEKAQAIMLIAAAAAAAA<br>ATKSSVATAVKPPMMPTTTVAPAAVVSPVLTRSPSLQSTSVATGQPQVVADPS<br>SICKLQADLPIARRHSLQRFLEKRRD

Table S4 Primers used for quantitative real-time PCR.

Gene ID	Forward primer (5′→3′)	Reverse primer (5′→3′)
ClJAZ1	GCCCAAGAAGGGCAAGAT	GTGCATGGAGCAGAGGTT
ClJAZ2	AGCGAAGGAAGAGGAAGGA	TCGTCGAAGACCACCATCT
ClJAZ3	CTGGCCAAGCGCAAGGA	ACAGCGCGATGGTGAGG
ClJAZ4	CATGGATCTCTTCCCTCAGAAC	CAAGGACTTTCCCACCATAGAA
ClJAZ5	AAGCGCCAGCTAACTATCTTC	TGAACCCTTGCTAGCCATTT
ClJAZ6	GCTGAGCCAGTACGTCAG	GCCGTACACGATGGTCAG
ClJAZ7	TTCATGGAGAAGCGCAAGAG	CAGTGGTAACAAGCAGCTGTA
ClJAZ8	CAGCGGTTCATGGAGAAGAG	CCAGGACCCTTGTTCTTGTT
ClJAZ9	CATTCTCGTTCCGTGTCTGT	CGTACACGATGGTTAGCTGT
ClJAZ10	ACAGGAGCATATTGTGGATGAG	CTGAGACGGACACGACATTATT
ClJAZ11	GCACTGGTGACACCGAATAA	GCCTGCATAGCTACCATTGA
ClJAZ12	GAAACGGCGTAAAGGTCAATTT	CCATTAGCTGGAGAGACACAAG
ClJAZ13	CCGTGAGAAGAGAAAGGAAAGA	CTCCCAGCAAATTGACCTTTAC
ClJAZ14	CGGTGCCCATCCATGTC	ACGAGGTGCTCCACTGA
ClJAZ15	CGAGGTCTATGTGTTCGACTC	GAAGCGGAGCTGATTGAATAAC
ClJAZ16	CAGGCTGCTGATGCTAAGAA	TTCTCAAGGAAGCGGTGAAG
ClJAZ17	CGATATGTTGAGGAGCGATGTG	GGGAGGATACGATCCAGTGTAA
ClJAZ18	CTGACTGATGCAAAGACCATAGA	CCACCAGCTATACCAGCATTAC
ClJAZ19	GGCGCAGTACATAGTTTCGT	CCAATCAAATTCCAAGCTCGTC
ClJAZ20	GCCTCCAATGATGCCTACAA	TTGCCCAGTTGCTACAGAAG
ClEF-1α	GGAAGTTTGAGACCACCAAGTA	CAATGATAAGCACCGCACAATC

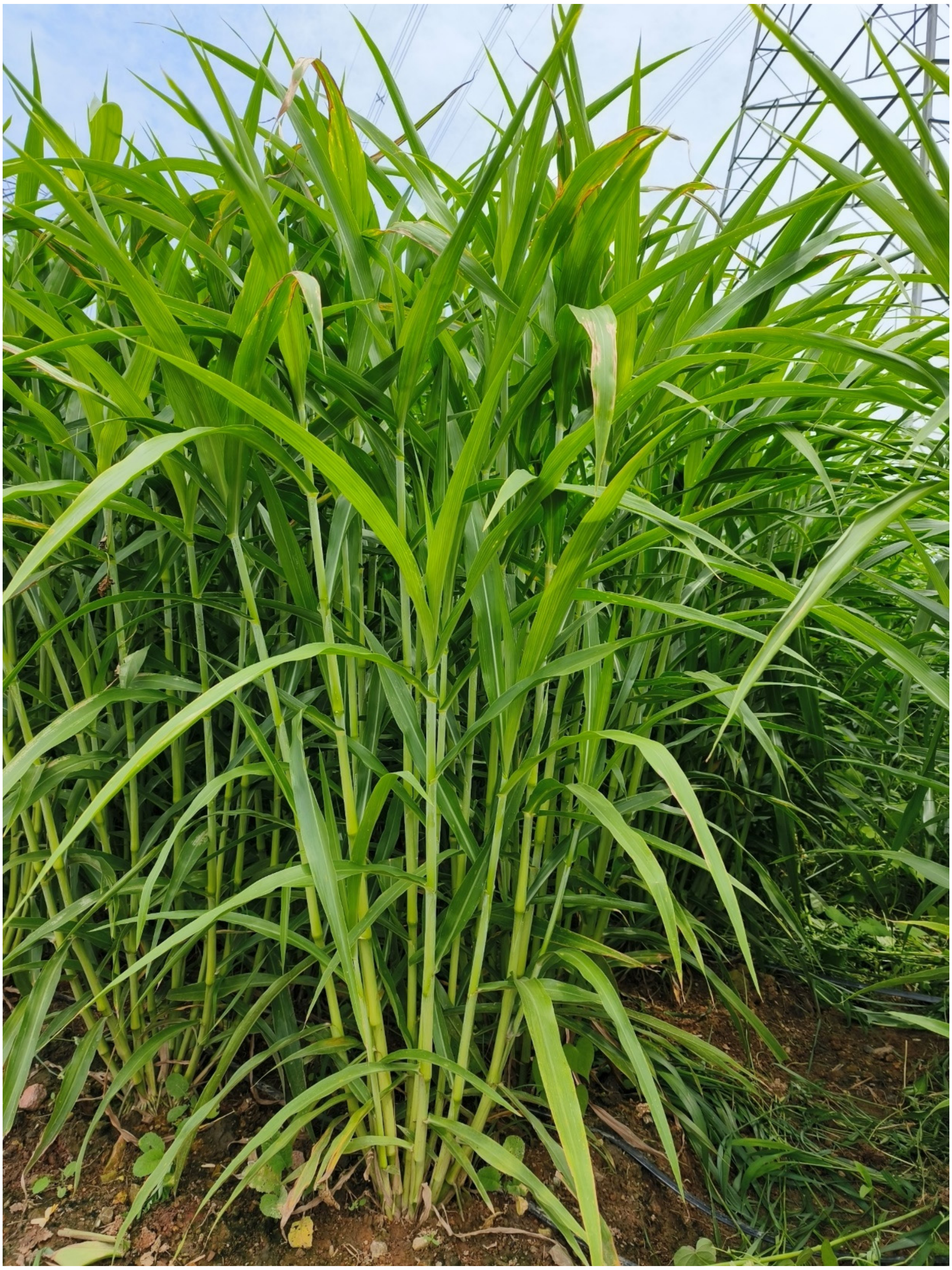

Figure S1 The cultivated *Coix lacryma-jobi* in seedling nursery of Hangzhou, China.

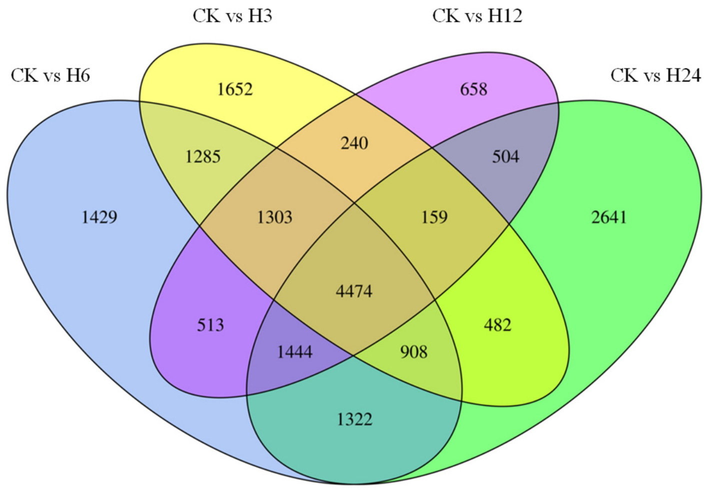

Figure S2 Venn diagram of differential expressed genes under control and heat stress. H3, H6, H12, H24 indicates heat stress for 3, 6, 12, 24 h, respectively. CK, control.

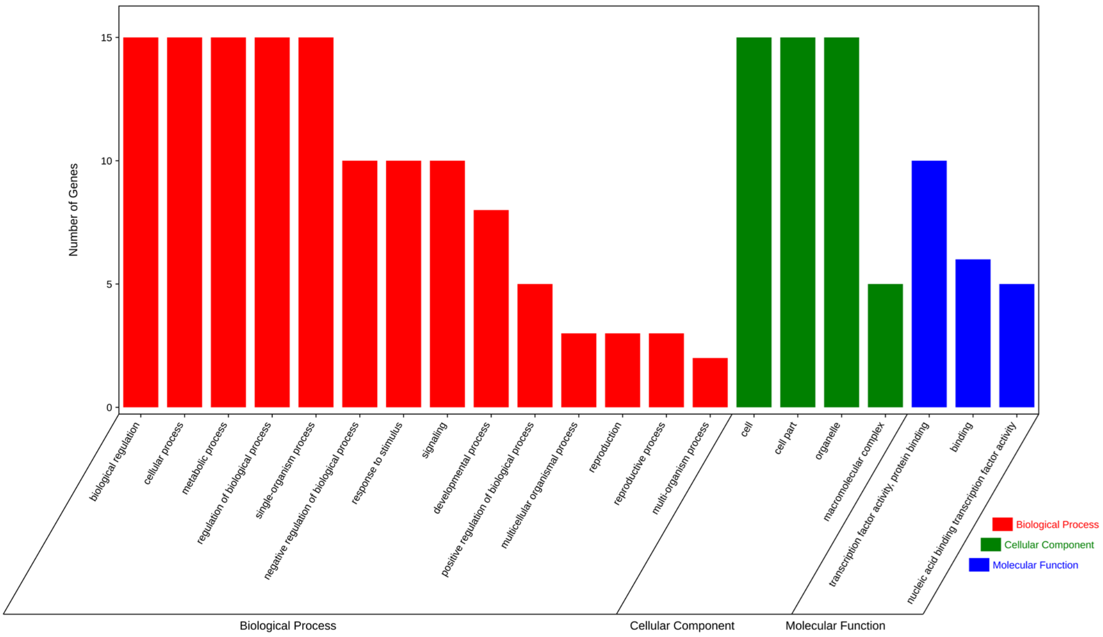

Figure S3 GO enrichment analysis of *ClJAZ1-ClJAZ20* genes in *Coix lacryma-jobi*.

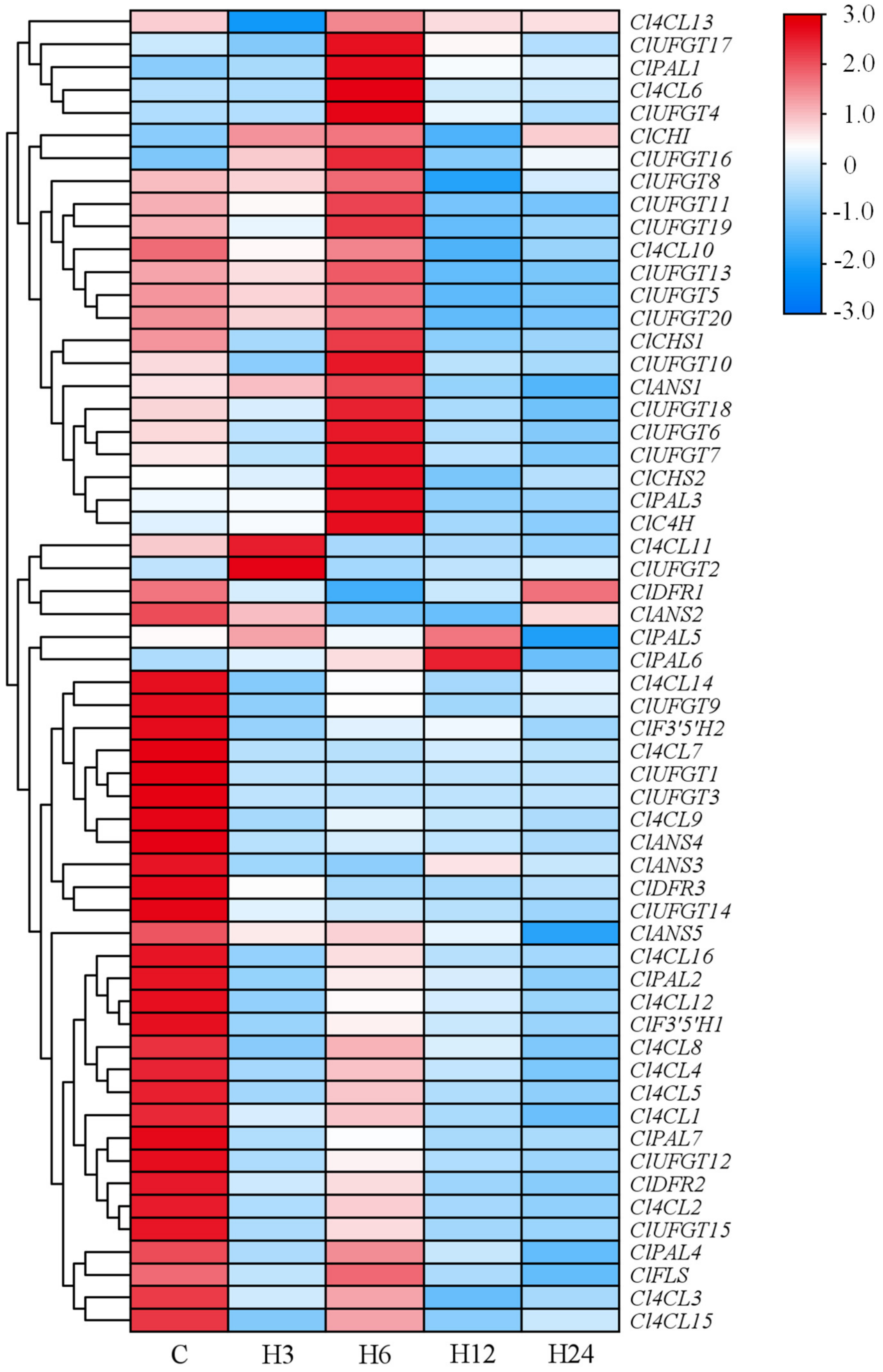

Figure S4 Expression profiles of flavonoid biosynthetic genes under heat stress. C, control. H3, H6, H12, and H24 indicates *Coix lacryma-jobi* plants exposed to heat stress for 3, 6, 12, and 24 h.
